# Cardiovascular disease training lessons learned during the COVID-19 pandemic: Need for change in training paradigm

**DOI:** 10.1007/s12350-020-02314-x

**Published:** 2020-09-10

**Authors:** Dakshin Gangadharamurthy

**Affiliations:** Cardiology Fellowship Program, Magnolia Regional Health Center, 611 Alcorn Drive, Corinth, MS 38834 USA

**Keywords:** Cardiology fellowship, COCATS guidelines, COVID-19, fellow in training


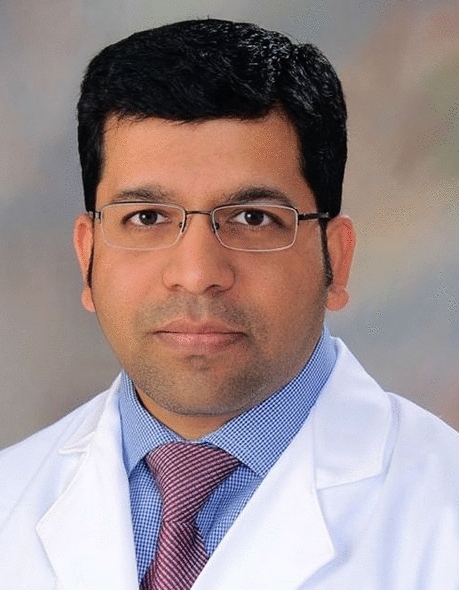
The novel corona virus (COVID-19) pandemic has led to an astonishing and unprecedented international health crisis which has resulted in exceptional challenges at the workplace and lives of the healthcare community. Patients with preexisting cardiovascular comorbidities are predominantly at high risk of developing complications and over 814,000 patients have succumbed to its overwhelming mortality across the globe as reported by the World Health Organization.

There has been a rapid surge of COVID-19 cases recently especially in Arizona, California, Florida and Texas with possibly a second wave of pandemic expected during the next winter. Federal government and the state authorities have taken aggressive measures to limit the spread of infection through social distancing.^1^,^2^ However, the reported mortality here in the USA is over 177,000 and has exceeded Vietnam War toll.^1^,^3^ Some of the common cardiovascular manifestations include but not limited to heart failure, arrhythmia, fulminant myocarditis, myopericarditis, and thrombophilia.^4^ Fellows in training (FITs) are at the forefront of patient care to meet the clinical demands of overwhelming influx of patients affected with COVID-19. The COVID-19 pandemic has led to extraordinary clinical demands on the healthcare community. In addition, it has affected the didactic activities and training experience of cardiology fellows significantly. Furthermore FITs with children, especially when both the spouses are in training are faced with unique challenges following the closure of schools and daycare which creates logistical problems related to work-life harmony.

## Changes Following COVID-19 Pandemic

The exponential surge of COVID-19 patients has influenced major reorganization in the structure of healthcare delivery. Cardiology fellowship programs are testing weeklong inpatient rotations for FITs on essential cardiology service to minimize risk of exposure to COVID-19 infection. Accreditation Council for Graduate Medical Education (ACGME) has allowed cardiology fellowship programs to self-declare pandemic emergency status lasting for 30 days, however, ensuring adequate supervision for training and educational resources in compliance with work hour policies of ACGME.^5^

Consistent with the institutional policies, fellowship programs have minimized the number of fellows in hospital to essential responsibilities such as critical care and consult service, night and weekend calls. All outside electives have been temporarily suspended or canceled. Several fellowship programs have maintained only essential rotations minimizing the total number of fellows in the hospitals at any given time. Outpatient clinics have moved to telehealth visits with provision for urgent in-person visits. Centers for Medicare and Medicaid services has introduced an array of temporary regulatory and reimbursement waivers with current procedural terminology codes to enable healthcare providers to offer additional services by telehealth visits.^6^ A standard cardiology fellowship generally comprises of rotations in acute hospital settings, noninvasive imaging, invasive procedures, electives and outpatient experiences for a well-balanced training.^7^ However, there are no standardized recommendations for cardiology fellowship training during the times of pandemic where education may not be a high priority. Several institutions have temporarily suspended or dramatically reduced hands-on procedures and educational experience to meet the demands for surge of COVID-19 patients. Although these structural changes present unique challenges, they provide several opportunities for learning. FITs are eagerly learning about cardiovascular manifestations of COVID-19 in real time. FITs assigned to non-cardiology services are challenged with infection control, airway management and use of experimental treatments. However, critical care experience and educational mile stones achieved through rotations in cardiovascular intensive care rotations will be helpful as the current reports indicate that COVID-19 infection and cardiovascular manifestations are closely linked.^8^ This gives an excellent opportunity for FITs to participate in pandemic planning meetings, design and development of clinical research related to COVID-19 and development of aptitude for leadership skills.

## Virtual Education

Centers for disease control and prevention recommends canceling large conferences and limiting meeting sizes. Our conventional model of live lectures and educational didactics has been significantly compromised.^9^ As a result, interruption of educational activities leads to poor learning experience. However, the educational strategies such as personalized learning, adaptive learning and flipped classroom proposed by American College of Cardiology (ACC) FIT leadership council could be helpful.^10^ A recent week long virtual nuclear cardiology elective held by American Society of Nuclear Cardiology (ASNC) was an excellent learning experience for FITs with large online participation and didactic discussion between FITs and the faculty. Such initiatives and promotion of virtual cardiology electives by ACGME through collaboration among ACC, American Society of Echocardiography (ASE), Societies of cardiovascular angiography and interventions, cardiovascular computed tomography and cardiovascular magnetic resonance may be helpful in the development of virtual electives to benefit FITs learning experience and training during COVID-19 pandemic and future natural disasters.

## Procedural Experience

As the duration of training is limited, a few weeks without procedural experience may have substantial influence on the development of hands-on skills and fellowship milestones. A recent study found significant reduction in the ST segment elevation cardiac catheterization laboratory activations in the USA during COVID-19 pandemic.^11^ Several institutions have limited the number of elective procedures such as trans esophageal echocardiogram, heart catheterization, device implantation and endomyocardial biopsy. While there is more flexibility available in large institutions, restructuring of rotations and simulations may strengthen the educational experience in small fellowship programs in compliance with ACGME policies. In addition, revision of core cardiovascular training statement (COCATS) guidelines may be necessary to meet the training requirements during COVID-19 pandemic and similar natural disasters in the future. Many institutions have implemented temporary hiring freeze in the wake of shortfall of revenue following COVID-19 pandemic leaving FITs in their final year of training with uncertain career outlook.

## Telemedicine

A drastic transformation of the healthcare system in the last few months has led to numerous challenges in the healthcare delivery for patients with cardiovascular diseases. Although there has been significant disruption in the midst of widespread COVID-19 pandemic, use of telemedicine has been instrumental in alleviating the shortfall of traditional clinical practice while limiting the exposure of healthcare providers to COVID-19 patients and limiting the use of personal protective equipment. In March 2020, Centers for Medicare and Medicaid services introduced reimbursement for telehealth visits at 0.7 relative value units (RVU) with an important caveat being the time spent by the attending physician on the phone, while the video visits will be reimbursed at a value on par with in-person visits.^12^

Many inpatient consults have also been transitioned to telemedicine video conference with patients and family to reduce exposure to COVID-19. At present, there are several health insurance portability and accountability act compliant telemedicine platforms available (Zoom, Doximity, Skype and Google Hangout). ACGME recently approved FITs involvement in telehealth visits with appropriate level of supervision. Although there are no specific guidelines, each should be tailored to meet the needs of individual patient, care teams and institutions. Some institutions have been using Zoom, Google Hangout and Doximity telehealth platforms for three-way conference with fellow and attending cardiologist on the line with patient. Currently there are no specific recommendations in terms of telehealth visits, however, with growing use of telehealth platforms and limitations of social distancing; revision of COCATS guidelines may be worthwhile during the COVID-19 pandemic or similar natural and man-made global threats.

## Changes to ACGME and COCATS Guidelines for Training

ACGME has mandated core competencies such as patient care, medical knowledge, practice-based learning, interpersonal and communication skills, professionalism and system-based practice to fulfill the requirements of cardiology fellowship training. Recently there has been a stronger focus on ambulatory, consultative, and longitudinal care. The curriculum also includes a requirement that continuity clinics be integrated with rotations in subspecialty fields such as heart failure, congenital heart disease, arrhythmia, and geriatric cardiology.^13^ These rotations may not be available in all the institutions under one campus. This may force FITs to seek training through outside elective rotations. However, in the wake of COVID-19 pandemic several institutions have imposed restrictions on outside elective rotations. These unforeseen changes may leave the FITs face the hardships in fulfilling the requirements. Hence, revision and changes to COCATS guidelines may be necessary to meet the requirements during COVID-19 pandemic and similar natural disasters.

## Challenges Faced by International Medical Graduates

Over 25% international medical graduates (IMGs) recently matched to 2020 cardiology fellowship training programs.^14^ IMGs are an integral part of the US health care provider workforce and IMGs have made exceptional contributions to patient care, clinical research and academia since the 1970s.^15^ About 40% of the FITs involved in treating cardiovascular disease patients are IMGs and IMGs play a pivotal role in caring for COVID-19 patients especially in rural and underserved areas of the USA. Furthermore, shortage of cardiologists is expected to increase during the next several years due to aging population and increase in the number of retiring cardiologists.^16^-^18^

On June 22, 2020, federal government introduced executive order suspending entry of certain nationals and categories until December 31, 2020 leaving FITs matched to fellowship programs on H1 and J1 visa in limbo.^19^,^20^ The matched FITs and fellowship programs are in legally binding agreement once they are matched as per national resident matching program (NRMP) guidelines. Failure to join the matched fellowship program within 45 days of start date of training is considered violation of NRMP match policies and the prospective candidates are barred from participating in the future NRMP match for the subsequent one to three years as per the NRMP policies delaying the fellowship training and career growth.^21^

**Figure 1:**
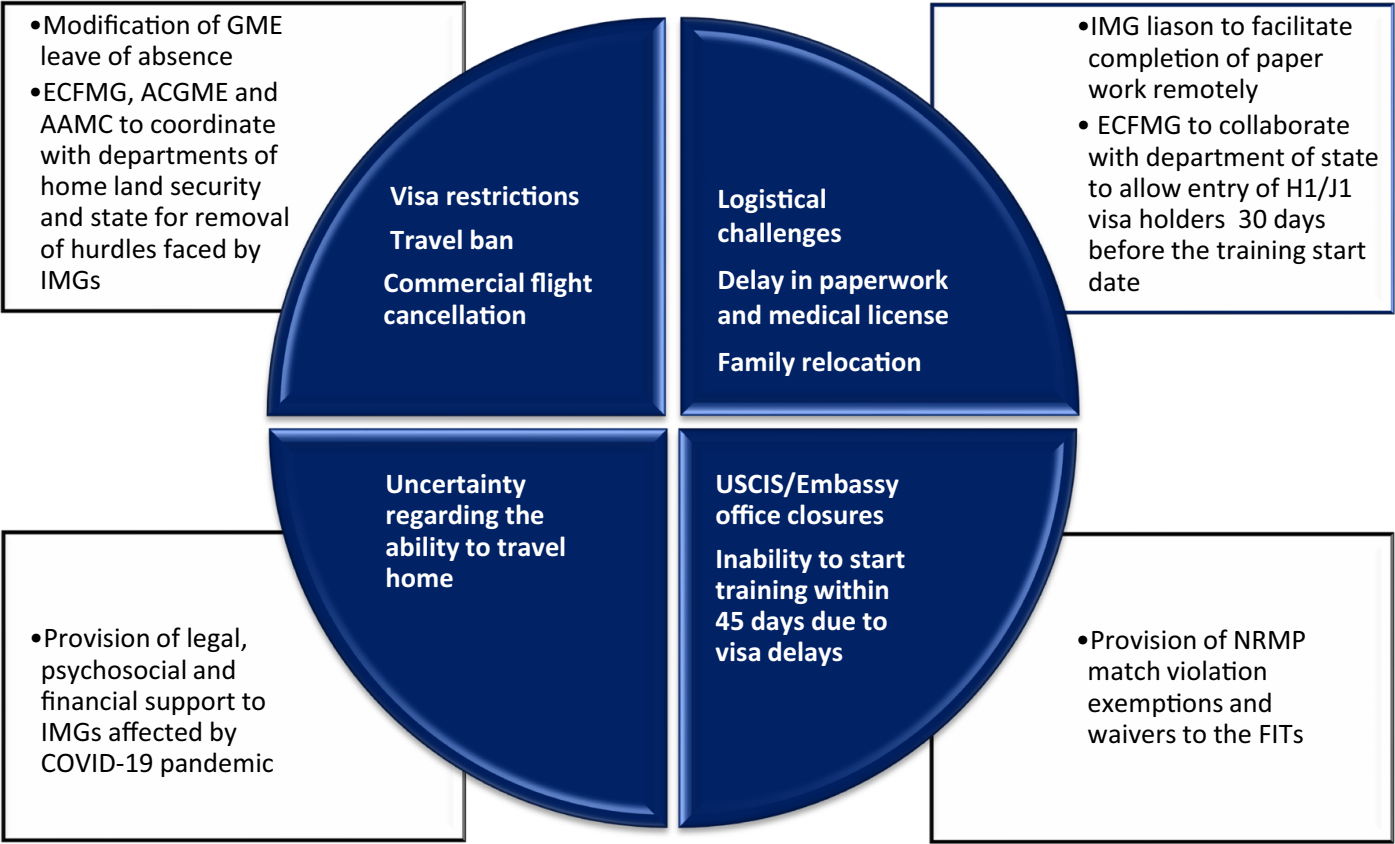
Challenges faced by international medical graduates and potential solutions

If the fellowship programs are unable to sponsor visa or FITs unable to procure visa, it would jeopardize both matched FITs and fellowship programs compromising patient care during the unprecedented times of COVID-19 pandemic. The timely transition of recently matched FITs to fellowship training programs is dependent on several bureaucratic, logistical, geopolitical, and factors related to COVID-19 pandemic (Figure 1).

## Potential Solutions and Proposal for Changes

1. COVID-19 pandemic has not only led to several limitations to fulfillment of traditional requirements of ACGME and COCATS guidelines but also significant disruptions to training experience of FITs and completion of educational milestones in a timely manner. However, some of these limitations could be overcome by online cardiology electives, virtual education/procedure simulation tools, innovative training tools and completion of such programs and credits towards ACGME milestones.
2. Traditional training methods underscore the emphasis on duration and volume based targets in procedures such as heart catheterization, trans esophageal echocardiogram and procedures of stress echocardiography and nuclear cardiology. Given the limitations related to COVID-19 pandemic and such possible natural disasters in the future, it may be worthwhile to revise the current ACGME/COCATS guidelines to meet the unanticipated training challenges in the form of creation of centralized/national digital library and repository of multimodality imaging studies, virtual and simulation tools of procedural training and didactics resources to be available for all the FITs enrolled in training. Promotion of intersocietal collaboration among ACGME, ACC, ASNC, ASE and Society of Cardiovascular Angiography and Interventions may be of paramount importance during the times of unforeseen international health crisis.
3. While larger institutions have adequate resources for FITs, smaller institutions traditionally send the FITs to outside institutions to fulfill the requirements of elective rotations. However, several institutions have eliminated or imposed restrictions on outside elective rotations to limit exposures to COVID-19 and to meet the clinical demands for surge of COVID-19 patients and unanticipated nature of patient volumes. Although these unexpected changes may cause disruption to traditional methods of elective rotations, FITs may collaborate with faculty and investigators of outside institutions in research activities. Collaboration and partnership among teaching institutions may help develop educational/training tools and promote platform for team work among FITs across institutions.
4. COVID-19 pandemic has presented several challenges; however, it may be helpful to identify ways to use the pandemic to our advantage as educational and research opportunity, substitute alternative ways of exposing FITs to procedures, consider using time away from canceled elective procedures for board review and scholarly activities and evaluate ways to promote sense of camaraderie and community during the pandemic and future natural calamities.
5. IMGs are an integral part of the US healthcare workforce and fellowship training programs. However, recent suspension of visa processing until December 2020 may lead to several hurdles to joining fellowship training programs in a timely manner. In addition FITs graduating from the training programs may face delays in transfer of their visa to prospective employers due to recent closure of several United States citizenship and immigration services (USCIS) offices/embassies following COVID-19 pandemic. Support and coordination among ACGME, USCIS, educational commission for foreign medical graduates and department of state are critical to improve the national physician shortage and meet the clinical demands of patient care in certain parts of the nation (Table 1).

**Table 1:**
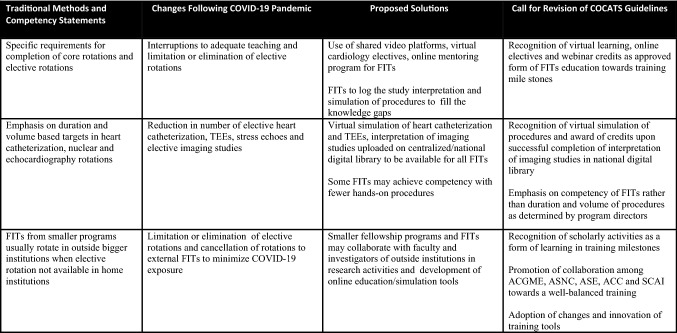
Potential solutions and proposal for changes

## Conclusion

The ongoing COVID-19 pandemic has exposed several potential shortcomings and limitations to cardiology FITs education. However, isolation has brought us together through social media and technologies with an emergence of innovative ideas. As Albert Einstein said “*in the midst of every crisis, lies great opportunities*”; COVID-19 pandemic presents several challenges and novel opportunities for FITs and the lessons learned during COVID-19 pandemic may give impetus to revise the COCATS guidelines to improve the training experience for FITs and be better prepared for the next natural or man-made global threats.

## Abbreviations

ACC: American College of Cardiology
ACGME: Accreditation Council for Graduate Medical Education
ASE: American Society of Echocardiography
ASNC: American Society of Nuclear Cardiology
COCATS: Core cardiovascular training statement
COVID-19: Corona virus disease 2019
FIT: Fellows in training
IMG: International medical graduate
NRMP: National resident matching program
USCIS: United States citizenship and immigration services
